# Dr. and Mr. Griffin on Functional Affections of the Spinal Cord and Ganglionic Nerves

**Published:** 1835-01-01

**Authors:** 


### Medico-Chirurgical Review,

### No. XLIII.

[No. 3 of a Decennial Series.]

OCTOBER 1, 1834, TO JANUARY 1, 1835.

### Observations on the Functional Affections of the Spinal Cord and Ganglionic Nerves, in which their Identity with Sympathetic, Nervous, and Simulated Diseases is illustrated. By *W. Griffin*, M.D. and *Daniel Griffin*, Esq. M.R.C.S. London, 8vo. pp. 247. 1834.

When we observed that one hundred and seventeen—or rather 148 *cases* were narrated in one small volume, it reminded us of the title of one of Sir Richard Phillips' books—a "Million of Facts." The impression, however, was not unfavourable, for, as our readers know, we are not among those who deride facts or cases, and pride themselves on the facility with which they can form theories and generalize, without the trouble of *particularizing*. In an investigation like the present, too, where so much intricacy, difficulty, and doubt exist, there needs no apology for accumulation of facts, however tedious or minute. The authors of this volume are entitled to the meed of great industry, at least, and we hope to shew that they merit much more than this. We shall proceed to the work of analysis, since we foresee a tolerable lot of labour in the task.

A long preface opens with encomia on Mr. Abernethy, the preceptor of Dr. Griffin, and with an opinion that, had that eccentric surgeon lived at a somewhat later period in the advance of physiological and pathological science, he would have attributed to irritation of the brain and spinal cord those functional affections which he attributes to "constitutional disorder," "nervous irritation," &c. Now that Legallois and others have shewn that different portions of the medulla spinalis form centres, from which nervous actions of corresponding parts proceed, and to which they tend, much knowledge is likely to accumulate by strict observation of morbid phenomena. Had Mr. A. (our authors remark) gone a little farther than he did, and acknowledged "that not only disorder of the digestive organs, but of the brain, lungs, or uterus, was capable of affecting the spinal cord (and thus producing sympathetic disorders of the body and limbs, without operating through the medium of the brain), he would have anticipated all that we could offer on the subject."

"The sensitive portion of the spinal cord and medulla oblongata seems obviously the medium by which derangement of the digestive or other organs act in exciting constitutional irritation, that is, act on the mind, on the vascular or secreting systems, or on the motor functions of distant parts; and again it is the medium by which distant local irritation, or the passions or affections of the mind, influence the digestive organs. To this reciprocal action and reaction Mr. Abernethy's doctrine is continually directed. For the most part, however, he overlooked the circumstance, that the spinal marrow is not always a mere inert nervous conductor between the source of disturbance and the distant affections indicative of constitutional irritation, but is itself the part absolutely and immediately thrown into a morbid state, of which these affections are but the symptoms; thus, when in disordered states of uterus or stomach, females of hysterical habits complain of pain of chest, cough, oppression, palpitation, and debility, these are not always to be looked upon as sympathetic complaints, occasioned we know not how by the uterine or gastric disturbance, but as signs of a morbid state of some portion of the spinal cord; of a secondary disease, in short, induced by the disturbance. It is the more necessary to hold this in view, as the morbid state or irritation of which we speak may sometimes occur apparently as a primary affection, or may remain as the sole disorder, when that which originated it has been long removed."—*Pref.* vii.

Our authors need not have apologized for using the term "irritation." It may not be so capable of definition as "inflammation," but it is just as necessary; and serves sufficiently well to convey ideas, which is all we want of terms and words in general.

"Though the relations of many parts of the system are still so mysterious, and the sympathies so complicate and extraordinary as to defy all explanation by nervous communication, much may in this way be understood, especially of the spinal cord and its connexions. Its physiology has been so very much elucidated by late experiments, that a little consideration would suggest to us the complaints likely to be produced by affections of any individual portion of it. From its continuity with the brain (that part to which intellectual actions have been assigned), and their known reciprocal sympathies, we should expect its diseases would excite many symptomatic disorders of that organ, as pain, vertigo, delirium, &c., which we shall find not uncommon. As the origin of all sensation and motion, we might anticipate simply painful, or spasmodic, or paralytic affections in any part of the body, or that general loss of feeling and motion in which we sometimes see persons lie, inanimate and powerless; the body still, the eyes fixed, the functions of respiration carried on imperceptibly, yet fully sensible of all that is going on around them. As it includes the origin of the fifth pair, which is found to be essentially necessary to every organ of sense, except sight, in the exercise of its functions, and even to sight to be accessory, irritation or disease near the trunks of that nerve should induce disturbance of the functions of the senses, or temporary paralysis of all or any of them, or painful affections of the extremities of these nerves themselves, as in the orbital, or facial, or alveolar branches. As the seat of the respiratory functions, we should be inclined to attribute to its disorder many complaints of the respiratory system. Near to the origin of the fifth are the roots of the respiratory nerves, the glosso-pharyngeal, the eighth pair, the spinal accessory, the phrenic; derangement or irritation of which should occasion affections of the throat, respiratory muscles, lungs, diaphragm, and stomach; loss or change of voice, hoarseness; crowing, croupy, or wheezing respiration; barking cough, globus hystericus, spasms of the chest or stomach, difficult deglutition, hiccough, weeping, crying, laughing, &c. Irritation at the root of the cervical nerves might induce, by communication, any of the foregoing symptoms, or occasion pain, stiffness, rigidity, or spasm of the muscles of the neck or arm, or numbness or paralysis. To irritation at the origin of the dorsal nerves we might attribute oppression, palpitation, pain in the anterior of the chest or stomach, or sides; and, at the origin of the lumbar nerves, abdominal tenderness, colic, constipation, pains in the loins, hips, extremities, with disorder or paralysis of the bladder, or of the lower limbs.

Lastly, the whole of the medulla spinalis, including the origin of the eighth pair, goes to form the ganglionic system of nerves. These supply all the muscles of involuntary motion, and all the viscera; and we have reason to suppose are mainly concerned in the secreting processes, among which Dr. Wilson Philip places the disengagement of caloric. They are distributed in networks round the arteries, perhaps solely supplying them with nervous power, and rendering them like the heart, though independent of, yet liable to be influenced by, any considerable portion of the brain or spinal marrow. In irritations of the whole or a great portion of it, therefore, we should anticipate irregular action of the heart, evinced in palpitations or in approaches to a suspension of all action, and syncope; interruption of the secretions, evinced in the oppressed breathing, failure of appetite, and flushings or burning heats, or universal shiverings, or coldness of the extremities or of particular members; and irregularities of the circulation, evinced in local, and often violent, determinations, or in loss of tone and vascular debility. How accurately these conjectures are borne out by subsequent cases, the reader will have an opportunity of considering." xi.

We are convinced, with our authors, that the present inquiry into spinal diseases will impress us with the necessity of examining more attentively the real dependence and value of symptoms. How ready are we, they justly observe, when the stomach is affected with pain, vomiting, and tenderness on pressure, "to conclude that there exists an acute or sub-acute inflammatory state of some portion of that organ, when, on a moment's reflection, we must admit that all these symptoms depend solely on the nerves, and may exist with or without inflammation—that, independent of the nerves, the stomach could neither have pain, tenderness, nor vomiting at all." The Broussaian doctrine could never spread so far and wide, if due consideration had been given to this subject, and if men had accurately observed how closely gastralgia will imitate gastritis, and irritation inflammation. Morbid anatomy is daily leading astray many thousands of our brethren, by inducing them to mistake effects for causes. Thus every tint of colour observed in the stomach, after death, has been put down to the account of inflammation, although these appearances are often wanting in genuine inflammation, and existing where no such disease could even be suspected. Dr. Yellowly's paper on the vascular appearances of the stomach, has been too little attended to by the Broussaians. One would suppose, from the writings of this school, that inflammation of the mucous membrane of the stomach was far more common than catarrh or bronchitis; yet such is not the case. The stomach being destined to receive an infinite variety of the most heterogeneous ingredients, Nature has wisely endued it with much less proneness to inflammation than almost any tissue in the body—and hence the abuse, which it daily and hourly sustains, from the excesses of the epicure and the drunkard, seldom wholly destroys its natural action, excites inflammation, or leads to organic disease. But we must proceed to the more immediate matter of the volume.

### Chap. I.—Introductory Observations.

Functional affections of the spinal cord are the chief subject of this introductory chapter. The singular, and apparently functional disorders which assume the garb of all others, and continue unrelieved by the remedies applicable to any which resemble inflammations, yet are exasperated by bleeding—which simulate spasm, yet defy opiates—and which sometimes eventually disappear of their own accord—are not a little puzzling, and are well entitled to attention. It appears reasonable to our authors, and to us, that some advantages may result from the attempt to trace nervous affections to one or more of the three great nervous centres; the brain, the spinal marrow, and the ganglionic system. All these systems are subject, independently of one another, to inflammation, to irritation, and the influence of sedative powers. The spinal cord itself consists of portions possessed of separate powers and functions—in other words, presents several centres from whence nervous energy is distributed to different parts of the body. Thus four of the senses derive their faculties from the superior portion of the medulla spinalis, including the medulla oblongata—independently of the brain or cerebellum. Its anterior part is the source of voluntary motion —its posterior, of common sensation. Between these is Sir C. Bell's column, which gives out the respiratory system of nerves. These considerations must lead us to question the propriety of attributing all nervous diseases to affections of the brain. There can be no doubt that a large proportion of these are attributable to irritation of the spinal nerves at their origins.

The authors do not pretend that their investigation into the nature of these irritations has led them to a successful practice;—yet surely if we ever hope to attain any thing like certainty in therapeutics, it must be through the medium of accurate symptomatology and pathology. We shall commence by laying before our readers an abstract of an extraordinary case, the first in the book, and the one which first directed the attention of the authors to the subject of spinal irritation.

*Case* 1. " A young lady, aged twenty-one, who had always before enjoyed good health, received a slight blow on the chest from her mother, during her convulsive struggles while dying of apoplexy. She spit up a little blood at the time, and felt pain for some days: after this it suddenly removed to the abdomen; affecting the left side, about the situation of the descending colon, and was accompanied by frequent pulse, tenderness, and the most incessant vomiting. The pain was abated by bleeding, blistering, and aperients; but nothing could allay the vomiting, which was brought on by the smallest quantity of any thing, solid or liquid, taken into the stomach. This came to be attended with flitting pains in the head, with throbbing of the temples, and intolerance of light, attributed to the straining; the continuance of which made it difficult to move the bowels. Even when medicine did operate, it gave no relief.

She remained many days in this state, suffering much from want of rest and the distressing retching; after which she was attacked with frequent oppression, occurring at intervals through the day, and usually terminating in fits of insensibility. In these she usually lay for ten or fifteen minutes, with her hands fast clenched, or sometimes shutting and opening them alternately with great rapidity. There was considerable rigidity of the tendons of the wrist, while the fit lasted; and the first symptom of amendment was always a gradual relaxation and opening of the fingers, when she fetched a long deep sigh, and recovered." 7.

These oppressions proved as intractable as the vomiting. Repeated blisters, bleedings, and exhibitions of half the Materia Medica produced no relief, or only the most temporary. After a few weeks, however, the more distressing symptoms began to give way independent of medicine, while the digestive organs improved in function by light bitters and aperients. She recovered so far as to be able to go out to parties and amusements.

This reprieve was only temporary; the oppression and cough returned, with pain in the left side. The cough was dry, loud, and convulsive, becoming, at last, incessant. "The convulsive expirations followed one another with such rapidity, that one could only conceive the suffering by imagining the fits of a severe chincough following one another without interval." The attempts to relieve the complaint were unavailing. At length the right lobe of the liver became enlarged, and formed a round, painful, and circumscribed tumor resembling a large abscess. Mercury was prescribed, and copious ptyalism induced. The cough was now relieved, and in a week or two ceased. As the soreness of the gums diminished, the cough returned with all its terrors. New symptoms displayed themselves, every week, or alternated with the old. Among other distressing symptoms, she was seized with severe pain and tenderness in the hypogastrium, followed by retention of urine requiring the catheter. There was scarcely any urine secreted for three or four days. During all this time the hard dry cough was incessant.

"The case was now looked upon as quite hopeless: the distress occasioned by such complicated disorder destroyed all rest and appetite, and induced extreme emaciation; solid food could no longer be borne, but was either instantly rejected, or excited violent spasmodic pain in the stomach, and sometimes the oppressions. The slightest motion (she was now continually confined to bed), brought on similar paroxysms, after which she usually became almost insensible, with suppressed convulsive efforts at coughing, her voice gone, and her pulse rapid. This state generally lasted for some hours, sometimes much longer; and, as strength gradually returned, the hacking eternal cough resumed its attack." 10.

It would be endless to enumerate all the phenomena which this unhappy lady presented. The disease successively imitated organic affection of the lungs, heart, and abdominal viscera. She lived almost entirely on milk, with a small quantity of fruit occasionally. Medicine was discontinued, as being thought useless. At the close of the year 1828, the spine was examined for the first time. There was no deformity, unevenness, or prominence of any vertebræ; but extreme tenderness of the whole column. Pressure on any of the spinous processes excited instant convulsive fits of coughing, with pain at the corresponding point anteriorly, or else oppression. The slightest curvature of the spine, in any direction, was intensely painful. "It seemed extraordinary how little the patient directed attention to the back in so intense a case of spinal disease." The complaint was now considered as clearly dependent on affection of the medulla spinalis, and all its various phenomena explicable on that ground. The extreme weakness, emaciation, and inability to turn in bed, prevented the application of blisters, and Nature was left to her own efforts. The complaint, however, increased—the whole spinal column became still more tender—all the phenomena exasperated in intensity. An issue was inserted on each side of the second cervical vertebra; by which the pain of the forehead, face, and scalp was considerably relieved; the other symptoms remaining *in statu quo*. At the end of February, 1829—

"While drinking in the evening, she felt a sensation as if something gave way in her chest, as if the band from the upper part of the sternum, before spoken of, had snapped. She was instantly attacked with oppression, a sense of burning and pain in the throat and chest, croupy breathing, total loss of speech, and blindness of the left eye, with numbness and paralysis of the left arm; she had also a sense of numbness extending from the point in the chest where she felt the band snap, across to the shoulder, and down the left arm to the fingers; some difficulty of swallowing, and violent pain, straining, or retching, when the smallest quantity of food or drink reached the stomach. There was some swelling and excessive tenderness of stomach, with violent cramp at intervals, which extended down to the limbs and knees. The secretion of urine was suppressed, no more than half an ounce having passed in twenty-four hours, and that thick and black. There was no tenderness or fulness in the pubic region.

After the lapse of some days, during which croton oil and diuretics had been freely used, the eye partly recovered its power, and the action of the kidneys was restored. Blisters to the throat and neck were of little advantage; but, on applying one to the occiput, some degree of voice was manifestly recovered, and the power of swallowing perfectly; the fingers of the paralysed arm also seemed to acquire a little motion.* In July, a very decided improvement had taken place. The arm had attained much strength; and she was able to speak in a low whisper, though with pain and difficulty. It should be observed, that the power of articulating was never lost, so that, even while partly dumb, she could often make herself understood by a distinct, voiceless articulation of the words." 14.

At the period of publication, this poor young lady was gradually improving in health, and entertained sanguine hopes of her own recovery. The phenomena detailed in the last quotation are mysterious, not to say inexplicable. It appears that "she now feels a sore tumor as if growing from the spine, and hitting against the sternum every time she coughs." We fear there is great mischief lurking behind yet; and wish that we may be mistaken. The sequel of the case, whether fortunate or fatal, will be very interesting. The case bears a close resemblance to one related by Dr. Monteith in Dr. Abercrombie's work, and a reputed similar one is next detailed by the authors themselves. We shall very briefly notice it here.

*Case* 2. A married lady, aged 45, was seized with acute pain and great tenderness in the ascending colon, with constipation, thirst, heat of skin, quick pulse, and vomiting. Bleeding, aperients, and blisters gave much relief—but the sickness of stomach continued incessant. The remission of the other symptoms lasted only two or three days, when they all recurred. The pain now extended to the transverse arch of the colon, with much pyrexia. Relief again was obtained by depletion. A third attack occurred, the sigmoid flexure of the colon being the chief seat of complaint. Depletion a third time. After several weeks of great suffering, there was found a hardness, or even enlargement of the liver; the evacuations being scanty

* "The paralytic attack seemed in the first instance to have affected the whole side; for although she never complained of the left leg, it was observed, in those convulsive thrillings of the frame which succeeded paroxysms of the pain and oppression, to remain perfectly still. It continued, however capable of the usual voluntary motions."

and black. Mercury was now prescribed, and copious ptyalism induced, "followed by a slow but progressive amendment." She eventually attained a tolerable state of health.

We have fairly abridged this case, and confess our inability to discover any very strong proofs of spinal disease in it. The authors themselves, indeed, do not appear to have suspected any such origin, till some time afterwards, when, on an attack "of violent spasmodic pain of stomach, followed by sickness, and violent pain in the back," the reporters examined the spine, and found "extreme tenderness of all the lumbar vertebræ, with a slight degree at the seventh dorsal." Our authors remark that the patient, unfortunately seldom complains of the back, in such cases, and hence the real nature of the malady is too often overlooked. In the following sentiment we agree with the authors.

"It should never be forgotten, that all affections at the sources of nervous power, or origins of nerves, are indicated by pain or disturbed action at the minute and distant extremities; and that this must hold true with respect to the brain and cord, as well as with any of the great nervous trunks in which the phenomenon is more frequently observed." 19.

*Case* 3. Bridget Leary, aged 22, complained of constant and distressing head-aches, with oppression and shrill piping noise in breathing—pains in all her joints—at the pit of the stomach—in the sides, hips, and limbs. These pains were aggravated by motion, and alleviated by the recumbent posture. On examination, the whole spine was found acutely tender. Pressure at the first or second vertebra occasioned pain, which shot forward from the occiput to the brow;—a little lower, pain was excited at the larynx. On pressing one of the lower cervical vertebræ, the pain occurred at the point where the trachea dips behind the sternum. On pressing the upper dorsal, the pain was felt at the middle of the sternum—from the third or fourth dorsal to the eighth or ninth, it was excited at the ensiform cartilage—still lower, at the sides, and in the lumbar vertebræ, the pain was excited in the iliac and pubic regions.

"Pressure behind the trochanter produced pain at the crista of the ilium, at the inside of the thigh, and also in the sides, or in the opposite hip. On the thigh or knee, it excited pain in the shins and toes. The pain was more acute on pressing the first or second cervical, and seventh or eighth dorsal, than any others; which accounts for the headach and pain of stomach having been the most constant and distressing of all the symptoms.

This is by no means either one of the most common or worst forms of spinal irritation. It may be of use, perhaps, to compare it with a case of chronic disease from injury, in which the anology seems sufficiently strong to assist or influence our views of its nature." 30.

*Case* 4. William Collins, aged 50, was caught up by the wheel of some machinery, two years ago, whereby his shoulders, back, and neck were much crushed and injured. He had a slow and imperfect recovery, remaining paralytic of the upper extremities, with contraction of the fingers, and debility of the lower limbs. He had also pain and stiffness in the muscles of the neck—in the limbs and joints—"and crackling noise on motion, as in chronic rheumatism"—frequent pains in the head, chest, and abdomen.

Examination of the spine, which was universally tender, gave the following results.

"Pressure on the first or second cervical vertebra occasioned pain over the brow; on the second or third, above and about the larynx; on the lower cervical, the lower part of the trachea as it enters the chest, and also at the top of the shoulder and in front of the chest. Pressure on the upper dorsal occasioned it at the superior part of the thorax; on the seventh or eighth, at the ensiform cartilage; on the tenth or twelfth, at the umbilicus. On the upper lumbar, at the sides and pubic region; on the lower lumbar and sacrum, at the groins, hips, and thighs. Behind the trochanter, at the knee and ankle." 21.

When only one point of the cord is affected, the symptoms are proportionably simple, and are more apt to deceive the practitioner, by their resemblance to chronic local affections of a constitutional nature. The following case is in illustration.

*Case* 5. N. Neville, aged 22, complained of pain at the pit of the stomach, of two months' duration. She has slight cough, with general languor and debility—head-ache—pain and stiffness at the back of the neck—weak appetite, &c. There was tenderness at the seventh dorsal vertebra, where pressure excited pain in the stomach. There was no tenderness of the cervical vertebræ. She was perfectly cured in a few days, by purgatives—blisters on the spine—and acid tonic bitters. In another case, where there was pain at the lower part of the sternum, and slight cough for two months, the bowels being natural, and the general health tolerably good, there was extreme tenderness about the seventh and eighth dorsal vertebræ, where pressure occasioned a darting pain towards the sternum, as if the patient were pierced with a sword. Being a labouring man, he would not submit to blistering; but he was cured, though more slowly than the former patient, by purgatives. Another case is mentioned of a man who fell backwards on a stone and injured his spine, about the seventh dorsal vertebra. On application at the dispensary, it was found that pressure on the spine occasioned instant pain at the pit of the stomach. He was ordered purgatives and fomentations. He did not return at the time appointed; and, on inquiry, it was found that he was so much relieved as to return to work. He dropped down suddenly and died.

They do not mean to deny that spinal tenderness is never a mere symptomatic affection. They take occasion to relate several cases where it was evidently symptomatic of intestinal, dental, or other irritation. "But even in many complaints of this kind, they observe, especially when existing for any length of time, the spinal affection becomes a serious and absolute disease, reacting on and increasing the disorder which gave it existence, or producing a new strain of symptoms proper to itself." In their early inquiries this was so frequently met with, especially in females, that they found it necessary to make a general examination of the patients attending the dispensary, in order to ascertain how far it was to be regarded as an independent affection. "The result shewed that it was very seldom wanting where those nervous symptoms supposed to indicate disorder of the spinal cord were present, and where there was no local disease to which it could be attributed—while, in almost every instance, in which acute or chronic local disease was found to exist, no such tenderness could be detected."

This is certainly strong ground for their doctrines to rest upon, if fairly and candidly stated—and we cannot question the truth of the statement.

"The minute attention to the spine which these examinations induced, led to much more interesting inferences than could have been at all anticipated. The great tenderness of the cervical vertebræ, in some cases of sudden fits of insensibility, suggested its existence in epilepsy,* in some forms of which it was found invariably present; a fact very well agreeing with M. Esquirol's dissections in this disease, which so frequently displayed morbid changes in the cord or its membranes. The connexion observable between tenderness at the same part and headach, soreness and pain of stomach, in cases of spinal irritation, occasioned the discovery of its existence in continued fever, in all cases where there was much disturbance of the stomach and head, and induced a suspicion that it was equally the source of Dr. Clutterbuck's cerebral inflammation and M. Broussais' gastro-enterite. The occasional occurrence of shivering fits in spinal cases pointed out some analogy between them and intermittents, and, as was conjectured, most acute tenderness of the whole spine was ascertained to exist in the very few of these complaints which fell within our observation during the last month. It was also detected in numerous cases of neuralgia, in many of paralysis, and in all that class of complaints called mimosa which came under our notice. In short we were finally driven to the conclusion, that the greater number of these disorders either wholly depend on some affection of the spinal column, or are strangely and importantly connected with it." 24.

The second chapter of the work treats of affections produced by irritation of the cervical portion of the spinal cord. Acute and chronic head-aches—brow-ache—pains in the cheeks, face, breast, side, sternum, shoulder or arm, are among the most common symptoms of cervical irritation. We shall advert to several of the cases.

*Case* 6. A young gentleman, aged 20, complained of intense pain in the crown of the head and forehead, with great soreness of the scalp, and general indisposition. He was subject to attacks of this kind, and was usually relieved by purgatives, and rest in the horizontal posture. "There was great tenderness of the five upper cervical vertebræ, pressure on any of which ocsioned pain in the vertex and brow." Purgatives and rest were again successful in relieving him. But leeches and blisters were recommended in addition.

*Case* 7. Ann Lynch, aged 19, was troubled with distressing head-ache, especially of the forehead, with sickness of stomach and thirst—pulse 95—tongue white—bowels constipated. Pressure on the first or second cervical vertebra, or behind the mastoid process, excited the pain severely at the brow. She was relieved by an emetic, followed by purgatives, and a blister to the neck.

*Case* 8. "Mary O'Brien, aged forty years, ill three years, complains of pain in the head, particularly severe over the brows and at the temples, and occasionally confining her to bed for days. She is very weak and nervous; has no appetite, and is worse after eating. Is occasionally attacked with pain of stomach.

* "When speaking of the diseases induced by irritation of the cervical portion of the cord, we shall give some most extraordinary cases of cure of this dreadful malady."

On examination, there was found extreme tenderness of all the cervical vertebræ, pressure on any of them, or behind the mastoid process, exciting the pain severely at the brow and temples. There was also soreness of the seventh or eighth dorsal vertebra, pressure on which occasioned pain at the ensiform cartilage. In this case there was so much general debility, and so many points of the spine were affected for a length of time, that a rapid recovery was not to be anticipated. She did well after some weeks, by the strictest attention to the digestive organs, a course of tonics, and occasional small blisters to the spine." 30.

Several cases are related, illustrating the various effects produced in different parts of the body by affections of the spinal medulla. Among these, the pain and sickness of stomach bear a prominent part. The stomach, indeed, is seldom free, and the head-ache is usually considered as merely symptomatic of the gastric affection—which, no doubt, it often is; but, observe our authors, much experience in these cases must convince us, that the sickness and pain, and loss of appetite, are still oftener themselves symptoms resulting from irritation at the origin of the par vagum.

" Various affections of the senses, loss of sight, hemeralopia, loss of hearing, noises in the ears, vertigo, spectra or visions, delirium and insensibility, are severally effects of irritation at the cervical portion of the cord, and are sometimes accompanied by headach of a very intense nature. But they are still more frequently met with where the spinal affection is general. Blindness, vertigo, deafness, ringing in the ears, are affections that scarce need particular illustration, appearing as occasional symptoms in almost every severe case which we may have to relate. Hemeralopia is more unusual." 33.

Examples of all these complaints are detailed, and some observations are made on the discoveries and experiments of Bell and Majendie, into which we need not enter. They remark that, next in frequency to affections of the fifth pair, from irritation of the cervical portion of the spine, are perhaps those of the par vagum. In many cases, indeed, the affections of these nerves alternate with one another—an event that might be anticipated from the proximity of their origins, the trunks of both growing from the floor of the fourth ventricle. The affections which they have ventured to connect with irritation at the origin of the par vagum are, sickness, vomiting, anorexia, inordinate hunger, pain at the stomach, pyrosis, and disorder of the respiratory system. We shall condense some of the cases, as they are very important in a pathological point of view.

*Case* 9. Michael Guerin, aged 16, received an injury on the back of the neck, about the fourth cervical vertebra. The blow was violent—he was put to bed—and seized with vomiting—tenderness at the epigastrium—hard, quick pulse, &c. He was bled, purged, blistered, and antimonialized. A degree of subsultus tendinum shewed itself on the third day, but subsided. Nevertheless, he lay in bed without fever, with clean tongue, and spirits undepressed; but could keep neither food nor drink on his stomach. In four or five weeks he recovered.

*Case* 10. A lady, aged 56, was seized with violent pain at the pit of the stomach and right side, attended with excessive soreness along the margin of the ribs back to the spine—constant nausea and vomiting, with febrile symptoms. The pain was periodical, like colic. She had also a stiffness in the muscles of her jaws, as though she was getting locked-jaw. The spine was examined, and there was found extreme tenderness of the lower cervical and upper dorsal vertebræ. There was also some tenderness of the lumbar vertebræ. The vomiting, which had resisted all other remedies, yielded to a blister to the stomach. "Irritation at the trunk of a nerve is sometimes unaccountably relieved by blistering the minute extremities, as Mr. Brodie has shewn, in the occasional benefit derived from blistering the knee in affections of the hip-joint." Her recovery was very slow, and frequently interrupted. It was effected chiefly by continued mild purgatives, anodynes, and rigid diet. She relapsed after this, and her stomach became so irritable, that she was obliged to confine herself to one slender meal a day—and even that was not retained long. She continued in this distressing state a long time, yet with scarcely any emaciation. The termination is not known.

*Case* 11. A patient in a house of industry was pointed out to the authors as not having been able to keep any thing on her stomach for five months. She was a young girl, 25 years of age, pale, weak, emaciated, and confined to bed. The catamenia had not appeared for seven months. No medicine had produced any benefit. On examination, there was found extreme tenderness of the second, third, and fourth cervical vertebræ, the slightest touch causing her to shrink and gather her features. There was no tenderness or enlargement in the hepatic or gastric regions, and the abdomen was much shrunk. As the vomiting was the most distressing symptom, a blister was applied to the cervical vertebræ. As soon as the blister had risen well, the vomiting ceased. The application, however, was renewed, and there was no return of the sickness.

Some cases of incurable organic disease, where vomiting was a harassing symptom, are related. The blistering of the spine relieved the sickness, though it did not, of course, arrest the organic disease. The following case is remarkable, and cannot be abridged.

*Case* 12. "A young lady, fair, and of delicate make, felt a gradual and uncontrollable languor growing upon her, attended by frequent headach and loss of appetite. After continuing in this state for some weeks, she was attacked with sickness of stomach and violent ineffectual efforts at vomiting, brought on by the slightest twisting or bending of the frame, or raising herself to the erect position. It was accompanied by swimming of the head and giddiness, with distressing headach, and followed by frequent fits of syncope, or insensibility, in which she used to remain for one or two hours, pale, cold, and apparently lifeless. She was sometimes affected with palpitations and great sense of sinking, with pain in the stomach and sides, especially the right, and extreme coldness of the feet; she had no appetite; the pulse was eighty; the skin cooler than natural; the tongue clean. The pain, sickness of stomach, and retching, were considerably relieved as long as she remained in the horizontal position; but the slightest motion of the trunk occasioned their instant recurrence, so that, when making the bed, it was found necessary to remove her in a recumbent position. She constantly complained of inexpressible languor. There was acute tenderness at the first and second cervical vertebræ, and it existed in a less degree in all the dorsal.

She was for some days treated by saline draughts, opiates, antispasmodics, and mild purgatives, but with little apparent benefit; the headach, giddiness, retching, and fits of insensibility, still continuing to recur on the slightest movement. A blister to the neck was then ordered, and there was immediate relief to all the symptoms; even the pain in the sides abated, and warmth returned to the skin. We could not learn that there had been any suppression or interruption of the catamenia; but it occurred on the next night, and the young lady was up and well in a few days." 52.

Our authors justly observe that preternatural hunger or thirst, occurring previous to convalescence, may be looked upon as symptomatic of some peculiar state of the cerebral substance, at the origin of the par vagum. The following case shews hunger as the first link in the chain of morbid symptoms attributed to spinal irritation.

*Case* 13. Mary Howard, aged 33, ill a month, complained of great hunger immediately after breakfast or dinner. Her stomach then became sick, but she did not vomit. Then came on pain in the epigastrium, lasting about an hour. The catamenia were regular, and the bowels were free—tongue white, pulse natural. On examination, there was found considerable tenderness at the second cervical vertebra, and, in a slighter degree, at the third; but none lower down. The stomach is painful on pressure. The treatment and issue are not mentioned; but the phenomena are confidently attributed (perhaps justly so) to spinal irritation. It would have been more satisfactory, however, had the remedies been stated, with their result. The following case, which we shall greatly abridge, is not a little curious.

*Case* 14. A delicate young lady had long suffered, at intervals, from oppression, constriction at the chest, hysterical paroxysms, and palpitations. She had tenderness of the cervical—of the middle and lower dorsal—and sometimes of the lumbar vertebræ. She sometimes suffered from neuralgia of the fifth pair of nerves, causing severe toothache. When this was relieved, there was generally pain or sickness of stomach, with oppression, palpitation, and pain in the middle of the back, or sometimes lower down. While labouring under an unusually severe attack, she was advised to have a blister over nearly the whole of the dorsal vertebræ. This occasioned a distressing degree of irritability and disturbance of the constitution, evincing itself by extraordinary hunger and thirst. In this state, some ale was given her, and she drank off a whole bottle in a few minutes, besides wine, demanding still more. She slept tolerably well that night, and next day came down to the drawing-room. She ate very heartily—took two glasses of wine before dinner—then ate broiled mutton, drank a bottle of ale, and said that nothing but wine and ale would satisfy her. An hysterical fit occurred, and was succeeded by a calm. She complained of pain in her side, which nothing but eating and drinking would relieve. She had eggs and ale for supper. "During that night, she got seven glasses of wine, and draughts of camphor julep." The mother prudently refused any farther supply, since she was "frightfully impatient, talking incessantly, and begging for wine and ether." Her stomach at length discharged its contents, together with bile, after which she was better. Next day, though the appetite was ravenous, the diet was restricted. It is necessary to state, that this young lady was previously unaccustomed to the smallest quantity of wine or ale. The inordinate sense of hunger and thirst, in these cases, is, our author thinks, connected with a feeling of nervous sinking, which is relieved by anything, solid or liquid, taken into the stomach. In such circumstances, it would not be safe to withhold stimulants altogether. Messrs. Griffin give some particulars of a case of unquenchable thirst, in a child affected with spinal irritation, and where four or five of the lower dorsal vertebræ were swelled and tender, with the usual symptoms of tightness, pains in the bowels, and general debility. Having taken some antimony for a cough, vomiting was induced, after which the most intense thirst took place, accompanied by spasmodic yawnings. Draught after draught was swallowed with the most surprizing avidity, and still there was the greatest impatience for more. During the attack, the pulse was too quick to be counted. Some antispasmodics, with ether, relieved her.

Passing over many cases, some from the note-books of the authors—others from periodicals, including one from this Journal, we come to a class of cases in which stupor or coma is a prominent feature.

*Case* 15. A girl, aged 14, complained of slight head-ache and heaviness for some time, but was not unwell in any other respect. One day, while reading, she fell suddenly on the floor insensible, but not convulsed. She soon recovered sense. She had three attacks of this kind within a short period of each other. On examination, great tenderness was discovered at the third cervical vertebra. Some blood was taken from the arm, and purgatives were exhibited, which prevented the attack for three months. On its recurrence, the same remedies were again resorted to; but in eight or ten weeks there was a fresh attack. A blister was now applied to the spine, and directed to be repeated at intervals. Nearly a year has now elapsed, without recurrence of the fit. More than once, however, there were threatening symptoms; but a blister occasionally, with purgatives, warded them off.

*Case* 16. Mary Cangney, aged 11 years, had fallen down frequently in fits, in which she lay for some time, with flushed face, speechless, and nearly insensible—generally from one to four hours. The pain in the head was instantly brought on by pressure on the cervical vertebræ—particularly on the second and third. Several of the dorsal were also tender, and pressure there sent a shooting pain up the neck and over the brow. Purgatives for six or seven days mitigated the fits, but did not remove them. Blood was then taken from the arm, and a blister applied to the nucha. The blister was neglected; but the bleeding gave entire relief for six weeks afterwards, when the fits returned in precisely the same shape as before. Bleeding and blistering entirely removed them.

" These cases are of exceeding interest, if only as illustrations of apparently formidable diseases yielding so instantaneously to the treatment. We may venture to infer from them, as well as from numerous others, how much more frequently even affections of the senses are the result of disturbed function than of organic mischief. These fits are frequently, as in the latter case, interrupted or cured by abstraction of blood. Whether this acts by relieving congestion in the spinal vessels, as many have supposed, or by quieting nervous irritation, we must not now stay to inquire. The attack is too frequently mistaken, especially in children, for a serious affection of the head; but is almost invariably at first symptomatic of some distant irritation, as of dentition, disordered stomach or bowels, or of a peculiar state of the uterine system; to which probably it was to be attributed in one of the foregoing instances. It is continually met with in all those spinal or hysteric cases in which the cord is affected to any extent; and though usually, and perhaps necessarily, connected with a deranged state of the cervical medulla, it seems sometimes to occur when the lower portions are more severely engaged; even so low as the middle lumbar." 69.

In the second section of this chapter, our authors take up the subject of "affections of the vascular system, connected with cervical irritation." It is reasonable to believe, what, indeed, experiments have proved, that although the heart, stomach, intestines, and other involuntary muscles, are wholly dependent on the ganglionic system for the exercise of their respective functions—yet that these same muscles and organs are subject to the influence of the brain and spinal marrow; and may be disturbed in their functions hourly by such influence. Several cases are detailed by our authors, illustrative of palpitation of the heart, angina pectoris, cough, &c. We shall glance at one or two cases.

*Case* 17. A labourer, aged 28 years, after severe labour in carrying loads of manure on his back, felt pain between the shoulders and in the back of the head, with continual drowsiness. He sometimes fell into a state of insensibility, in which he lay some minutes. He lost all appetite, and was frequently attacked with racking pains or cramps in the stomach and sides. After some weeks, the head-ache became distressing, accompanied by palpitations, tremblings, or shiverings, and universal throbbing of the arteries. "He felt pulses in every part of the body." The palpitation often occurred in sudden paroxysms, the heart beating as if it would burst the walls of the chest. It frequently extended to the descending aorta, and was most distressing at the back. When attacked in this way, he was always obliged to spring from his bed, and walk about the room till the throbbings ceased. After some months, his head-aches became so distressing that it was necessary to bleed him largely from the temporal artery—with considerable benefit. But the palpitations continued, usually preceded by general rigors, followed by pains between the shoulders, profuse perspirations, &c.

The treatment consisted of bleeding—the constant use of antimoniated ointment to the spine—the compound aloetic and assafœtida pill, &c. and one or two blisters to the back of the neck. There was a perfect recovery in three or four weeks.

"It is necessary again to call the reader's attention to the spinal tenderness which, from its almost invariable presence in such cases as occurred to us, we believe may be found in the very earliest stages of many of those dreadful fits of palpitation, angina, syncope, &c. dependent on dyspepsia and hysteria. It can matter little, whether it be characteristic of a primary affection, created we know not how, or of one originating in the mental, the digestive, or generative organs, if it is capable of becoming independent, and reacting on the system. The only question of importance appears to be, is it occasionally or ever induced by organic disease of the heart or large vessels? or is it not rather the precursor of these?" 79.

Our authors observe that it may appear paradoxical in them to suppose, that functional disorder can imitate the *physical signs* of organic disease. What are "physical signs" but mere *symptoms?* Palpitation of the heart —bruit de soufflet, &c. are called physical signs; but they are mere symptoms, which functional disorder can imitate. It is the visible *post-mortem* changes that they cannot imitate. Laennec acknowledges that many of the organic diseases of the heart—or, at least, their outward physical signs—as bruit de soufflet—may be imitated by functional disorder.

"With cases of violent, or irregular, or depressed action of the heart may be considered similar affections of the arterial system, which will very generally be found to depend on that morbid condition, of a greater or less extent of the cord, called irritation. Preternatural throbbing of the temporal and carotid arteries will usually be found connected with cervical tenderness, while the pulsations of the aorta in the epigastric region are more probably referable to irritation of the upper dorsal. We speak in this qualified way, because, in all the cases we have met with, there existed tenderness of the whole or the greater part of the spine. Before functional disorders of the cord became a subject of investigation with us, we were often much alarmed and perplexed by these epigastric pulsations; and, in cases presenting many marks of an exquisitely nervous diathesis, were imperfectly contented to assume, with Dr. Baillie, Hunter, and others, that they were not dependent on organic disease; but we now have, it is hoped, in the spinal tenderness, a certain diagnostic, by which apprehension may be at once allayed." 81.

Our authors have had every reason to coincide with Laennec in viewing angina pectoris as a neuralgic affection; and, as far as their experience goes, it is connected with spinal tenderness--whether of the cervical or dorsal vertebræ—or of both. We shall condense a case in illustration.

*Case* 18. A lady, aged 45, had suffered for some years with violent head-ache, especially of the brow or forehead—generally worse in the right than left side—attended with throbbing of the temporal arteries, flushed face, and feverishness.

"The attack comes on in rather a singular manner: her vision first seems suddenly dim or troubled, or the half of any object she looks at disappears. If her eyes are fixed on the window, the glass appears to move like water flowing in sunshine; or, if engaged in reading, one half of the letters seem wanting. This is an infallible precursor of the pain; and, as soon as it occurs, she gives up whatever employment engages her at the moment, and prepares to meet it. He stomach soon becomes sick, and this is followed by the violent pain in the head, which continues an uncertain period, from a few hours to three or four days. Her right eye is usually affected first, but eventually both. Her general health appears good; her habit inclines to fulness." 82.

She had been ill some years before with constant palpitation and nervous catchings or startings, when in bed—oppression—pain in the chest, arms, neck, and stomach. By one physician it was considered as organic disease of the heart—by another as hysteria. The latter physician cured his patient by aperients and the usual remedies. In an attack like that described, our authors were consulted, and applied leeches to the temples, with active purgation, by which the attack was removed.

"About twelve months after, we were called up in the night to this lady, who said she was dying. It appeared, that, some hours after retiring to rest, she was suddenly awakened by violent palpitation, with a shooting pain in the region of the heart, extending from thence down the shoulder and arm: she started out of her sleep, pale and terrified, and felt as if she was about to die; but in a short time the pain lessened, and the palpitation began to subside. On arriving at the house, she appeared much relieved; but a feeling of languor and apprehension remained. As our attention was at this time beginning to be directed to functional affections of the cord, the cervical vertebræ were examined, but no tenderness was detected. The dorsal were not examined at all. She was treated with aperients, camphor, and other antispasmodics; but the palpitation continued to recur for several nights, though in a much less degree. As it subsided, she began to complain greatly of the back of the neck: the muscles were so sore that she could not bear the gentlest friction, nor could she turn her head or stoop without pain: there was also pain round the throat, and down either shoulder: she had uneasiness in the neck when this attack first came on, but her terror at the palpitation prevented her attending to it. She said, when examined, that the pressure was not made low enough. There was now excessive tenderness of the lowest cervical vertebra. It did not still occur to us to examine the dorsal, as we connected the symptoms in our minds exclusively with irritation of the cervical or pneumogastric nerves." 83.

In the following Summer they were again summoned at midnight to this lady, she having another paroxysm. Her breathing this time was shrill and stridulous, threatening suffocation, and resembling spasmodic croup. The paroxysm, however, was nearly over when they arrived, and on examination, there was found extreme tenderness of the first, sixth, and seventh cervical vertebræ. She speedily recovered from this attack, by the usual means: but a few months afterwards, had an attack somewhat different—viz. pains in the hip, knee, and ankle, coming on in paroxysms of intense severity, darting down the limbs and attacking the joints severely, without heat or fever. This was considered a true neuralgia, and was readily relieved. She would not, however, persevere in applications to the spine, and was therefore likely to have relapse.

There is much truth, as well as force in the following observations:—

" Previous to the time of Corvisart, those resulting from organic lesion were little understood, and believed to be very uncommon. The most obvious cases were treated as nervous disorders. The study of morbid anatomy at length produced a total change in medical opinion; and it soon became a universally-received notion, that most or all of them were connected with absolute physical alterations in the organs affected. As a consequence of this, numerous patients who were suffering from dyspepsia, gout, or rheumatism, were put under the severest discipline, and thrown into a miserable state of dejection, by pronouncing their cases organic. Frequent error has now led us to tread back our steps; and we would only beg to remind our readers, that the stronger the necessity would seem for doing so, the greater must be the risk of our again, like the older physicians, overlooking organic diseases. We know that those called nervous or symptomatic are infinitely the most numerous; but we are also fully impressed with the conviction, that instances of altered structure are not of rare occurrence." 85.

In the third section of this chapter our authors take up the subject of "affections of the respiratory system connected with cervical irritation." Cough is one of the most common—and one overlooked by our best writers. It occurs in many forms, and connected with a variety of symptoms—sometimes hard, dry, and constant—or coming on at a particular hour in convulsive paroxysms, with or without fever—generally accompanied by gastric, hepatic, or uterine disorder. In the majority of spinal cases it appears as a common catarrh, but without expectoration.

" Spinal tenderness may be found in all these cases; and, as has been so often stated, may keep up the symptoms after the cause in which it originated has been removed. Mr. Burns seems to apprehend, that, in young females, if the spinal affection is overlooked or neglected, consumption may be induced. That irritation at the trunk of the respiratory nerves may induce tubercular inflammation as readily as when acting on their minute fibrillæ, in the bronchial membrane, there can be no possible doubt; but this can only be admitted with respect to cases in which a phthisical diathesis prevails. Numerous instances have occurred to us, in which most harassing cough was kept up for months, and even years, by irritation of the cervical portion of the medulla, without inducing any more formidable disease. One case only we recollect to have terminated in fatal consumption; but this was very protracted; and all our entreaties could not induce the patient to persevere in attention to the upper part of the spine, although the most marked benefit, and at one time an interruption of the disorder for months, was procured by repeated blistering: she could not be persuaded that remedies applied to the neck were of avail for what she conceived an affection of the lungs. Indeed it seems very probable, that, in many cases of hereditary phthisis, the disorder commences in this way, and might often be prevented by vigilant attention. Cough may always be excited in these irritations by pressure on the tender vertebræ, and sometimes convulsive fits, or hard barking occurs, from any unusual action of the diaphragm, or accidental excitement of the mucous membrane of the lungs. Thus, a sudden fit of laughter, or of crying, may bring on coughing for hours, or it may be occasioned by a stimulant vapour, or by mere mental excitement." 87.

We have no doubt that the foregoing passage and the cases appended to it, will lead many practitioners astray. No day passes without our seeing the dangerous errors of mistaking real for symptomatic affections of the chest. The patient is always prone to assist in the false diagnosis, attributing the cough to stomach, liver—any thing except the right one! In this way thousands are daily going to ruin, swallowing mercury and a host of remedies for indigestion, hepatitis, &c. instead of having repeated leeches and blisters to the chest, with confinement to the room, or even to bed. Putting our junior brethren on their guard, we shall proceed with some of the cases.

*Case* 19. "A young lady, aged seventeen, of delicate frame, light-coloured hair, and peculiarly fair skin, was attacked with cold; which left a short, slight cough behind it, not very troublesome, but occurring frequently in the day, and accompanied by slight oppression. The oppression was greatly increased on ascending the stairs, and there was then some palpitation. The cheek was always coloured with a pink spot, beautifully defined and bright, especially in the morning. The pulse was quick and readily excited, usually 120 in a minute; the tongue was whitish towards the middle and back part, and the papillæ elevated; she had tenderness at the lower part of the sternum, and often pain there on deep inspiration, and complained of general languor and weakness. There was tenderness of all the cervical, and of the four upper dorsal vertebræ; pressure on any one of which instantly brought on coughing.

The treatment in this instance consisted in the application of ten leeches, and long narrow blisters, often repeated, to the tender part of the spine. Minute alterative doses of the blue pill were also given, and a mixture composed of infus. rosæ, with Epsom salts and tincture of digitalis. The most evident amendment followed the blistering. In proportion as the spinal tenderness abated, the pulmonic affection gave way, and she was quite well in a fortnight or three weeks, although the attack had existed for a considerable time previous." 87.

The above, we acknowledge, is a strong case; but still we have our doubts respecting its spinal origin or cause. It must be remembered that the young lady was necessarily confined to the house by the blisterings and leechings, while the saline aperients and digitalis all tended to reduce inflammatory action wherever seated. The same methodus medendi, in fact, would have cured bronchitic inflammation, whether there was spinal irritation or not.

And why did not our authors apply the stethoscope and prove the point at issue. The following case is given by Messrs. Griffin, by way of contrast to the foregoing.

*Case* 20. A lady, aged 17 years, became affected with pain in the right side, with great tenderness on pressure, nausea and feverishness. The latter symptoms were removed by purgatives; but the pain continued, varying its situation slightly—but sometimes shifting entirely to the left side, or both at the same time. Nervous and hysterical symptoms soon came on, with oppression, head-ache, and eventually, a dry, loud cough, with disposition to chilliness and shivering. The pulse was usually 120—catamenia regular. Great tenderness was found about the second cervical vertebra, pressure on which occasioned acute pain in the vertex and brow. Pressure on the lower cervical and upper dorsal excited pain there, and loud coughing—at the seventh or eighth, the same symptom, with pain of chest—and, at the four lower dorsal, there was extreme pain on pressure.

"Leeches and repeated blistering at the tender points of the vertebral column were employed, with great temporary relief; but, when the severity of the symptoms abated at one part, it increased at another, as if there had been rather a transference than an actual removal of irritation. Thus, she sometimes complained most of the head and stomach; sometimes of the distressing cough, pain of chest, and oppression; sometimes of the back and sides. A considerable amendment took place during the use of mild purgatives, followed by tonics; and the cough seemed at last to yield to the sulphate of quinine." 88.

All the symptoms, however, recurred in an aggravated form a few weeks afterwards, in consequence of some mental affliction—the cough, pain in the side, &c. grew more distressing than ever. She was put on a course of mercurial frictions, which removed the cough in a fortnight, after which the general health improved. Still she had another relapse a few weeks afterwards, which was removed by purgatives with tonics, and small doses of opium, stimulating liniments to the spine, &c. Her recovery is stated to be perfect.

Messrs. Griffin confidently conclude that the hard barking cough described by Sir Charles Clarke as affecting young females, and often cured by sudden affusion of cold water, is merely a symptom of spinal irritation, and that spinal tenderness would have been found in all the cases described, had examination been made. Next in frequency to cough is oppression, varying from slight dyspnœa to the most terrific paroxysm of asthma. Various cases are related, but we have not space for their details. They think that spasmodic croup is, in most instances, dependent either on irritation at the origin of the eighth pair, or at some part of the fourth ventricle. Three cases of this affection occurring almost at the same time, confirmed our authors in their views. They strongly advocate the existence of spasmodic croup, as a distinct disease, and different essentially from inflammatory croup. They accordingly take some pains to controvert the opinions of Drs. Cheyne and Kellie, who maintain the identity of the two affections. Few observant practitioners, nowadays, doubt the occasional existence of purely spasmodic croup.

In the fourth section Messrs. G. treat of affections of the motor system, as connected with cervical irritation. They observe that there is probably no form of disorder produced by change of structure, which *irritation* of the same structure may not imitate:—and if chorea, convulsions, epilepsy, trismus, and tetanus can be excited by *organic disease* of the spinal marrow, they may be equally so excited by mere irritation. In some cases of epilepsy our authors made examination of the state of the cervical portion of the spine, and found extreme tenderness there. The cases were of long standing—one of them fifteen years—and yet the facility with which they were relieved must remove all doubt about their being functional affections. Three cases are related in illustration. We shall make room for one.

*Case* 21. " Soon after the occurrence of these cases, a young woman while waiting in the Dispensary for medicine for her father fell down in an epileptic fit, and was violently convulsed. On her recovery, she said she had been subject to these fits for fifteen years, occurring sometimes three times a day. She had done nothing for their prevention for many years past, conceiving it perfectly useless. All through she had at times suffered from pain in the brow, stomach, abdomen, and groins. On examination it was found all these pains were successively brought on by pressure at the corresponding parts of the spine; the tenderness was considerable at the three upper cervical vertebræ, and the eyes looked heavy. Blood-letting, purgatives, and blistering were employed, as in the foregoing cases, with immediate and perfect success. Nearly twelve months are now gone by without the occurrence of a single fit, although it was almost of daily occurrence for fifteen years. This patient was about twenty-nine years of age, and had the catamenia regularly. As the tenderness of the vertebræ had a constant tendency to recur after the blister had healed, she was directed to rub small portions of tartarized antimonial ointment frequently along the spine, so as to keep up a constant counter-irritation there; and she found so much benefit from this plan, that she continued for some months of her own accord, pursuing it strenuously whenever she was attacked with pain in the brow or with other threatening symptoms." 112.

Since the above cases occurred, they have met with some others, chiefly in men, where there was no spinal tenderness, and where no remedies had the slightest good effect. It is probable, therefore, that the convulsions in the female cases were more of an hysterical than true epileptic character. Cases of paralysis, chorea, convulsions, tetanic spasm, &c. are given, but we must refer to the work itself for their details.

### Chap. III.

This is a very short one, and treats of affections resulting from irritation of the dorsal portion of the spinal cord. These affections are almost wholly confined to the superior extremities, and upper portion of the trunk. They consist, like those already described, of pain or loss of sensibility—in convulsion or loss of power:—or they may appear in the form of general disturbance of function in particular organs. Among the first class we meet with pains in the collar bones, shoulders, arms, &c. often mistaken for rheumatism—or at the middle or lower part of the sternum, supposed to be affection of the lungs, especially if accompanied by dry cough—or in the female mamma, leading to the apprehension of cancer. Again, we find pain of side, just below the right or left breast, frequently exciting suspicions of phthisis—and in the right hypochondrium, simulating liver affection. Lastly, we have gastrodynia, and sense of tightness at the epigastrium.

The attacks in which loss of sensibility is the prominent feature, are those of numbness, and perhaps dyspnœa—loss of power or partial paralysis of the arm or hand—or, when affecting the intercostal muscles, in a sense of weight or oppression at the chest. Among the disturbed functions of particular organs, may be noticed some affections of the lungs, diaphragm, and stomach, already alluded to—cough, oppression, spasms of the bronchial tubes or asthma, hiccup, uncontrollable fits of laughter and sobbing, altered gastric secretions, flatulence, eructations, sense of distention, &c. Though these may be strictly disorders of the phrenic and pneumogastric nerves, they seem to be sometimes connected with spinal irritation.

"There are but few points, on which it is worth while to offer a remark with respect to these affections. As they often set in, or are attended at a very early period by pain and tenderness in the right hypochondrium, and are sometimes relieved by mercury, it becomes an interesting question, whether they have originated in disease of liver, or whether the pain and tenderness are truly nervous, and relieved only by that specific action which it would seem mercury exhibits in some diseases of the cord as well as of the liver. It would also appear very deserving of inquiry, why extreme tenderness, with the corresponding pain of stomach, is so much more frequently met with in the situation of the seventh or eighth dorsal vertebra than at any other point of the spine. Dr. Brown attributes it to the great degree of motion which takes place there; but, if that were the case, we should have it at least as often in the upper lumbar, and much more frequently in the cervical portion of the column, which is in continual motion. Yet this is by no means the fact. Tenderness about the eighth dorsal vertebra is almost always met with in the worst forms of spinal irritation, is seldom absent in the mildest, and sometimes exists in itself the sole malady. It is continually found a prominent and troublesome symptom in advanced pregnancy, in cases of uterine disorder, in derangement of the digestive organs, and in mental affections in females of hysterical habits—constituting this portion of the spinal marrow, as it were, the sensitive centre, to which most functional affections of the system are in the first instance referred." 124.

The cases themselves we are compelled to omit. They are sufficiently illustrative of the foregoing text.

### CHAP. IV.

This is on affections produced by irritation of the lumbar portion of the cord. The authors conceive that functional and organic affections of the kind in question were continually confounded together before the late discoveries in physiology, although a few talented observers had begun to detect and distinguish them previously. Mr. Abernethy pointed out the existence of a disease simulating an affection of the vertebral bones, and yet not of that nature; Sir B. Brodie long ago published cases resembling caries of the hip-joint, or ulceration of the cartilages; yet where the complaint was hysterical. Dr. Gooch, too, gave an interesting account of painful complaints of the uterus, unconnected with organic change. These and many others, are included in the class neuralgia, are generally looked upon, either as idiopathic, or as symptomatic of irritation in some distant organ. "They are seldom attributed to the source we are endeavouring to trace their connection to—disturbance of the spinal marrow."

"We may consider these disorders, like those of the cervical or dorsal portion of the cord, as consisting in preternaturally increased sensibility or action, or in a diminution or loss of either. Among the former, we have pains in the sides or abdominal parietes, colic, pain in the kidney or bladder, or uterus or ovarium, or in the spermatic cord or testes, pains in the joints or muscles resembling rheumatism or ulceration of the cartilages of the knee or hip joint. Again, we have cramps in the bowels or legs or feet, or we have diarrhœa, leucorrhœa, or menorrhagia. Among the complaints marked by loss of sensibility or power, is a sense of weight or fulness of the abdomen with flatulence, and, perhaps, obvious distention. This would seem to depend on loss of sensibility in the intestines themselves, and is generally connected with obstinate costiveness. It is the state sometimes induced by the administration of the carbonate of lead. There is another of the same nature in which the spinal nerves are those chiefly affected, denoted by diminished sensibility of the abdominal muscles. Such is the case with persons who feel as if their bowels were falling out, or with those who feel like the gentleman described by Mr. Abernethy, as if they had no bowels. We have also in the same class defective or suppressed secretions, and partial or total paralysis of particular muscles or organs." 129.

Many illustrations are appended to this statement—partly from the practice of the authors themselves, and partly from the writings of others.

### Chap. V.

This treats of "General Irritation of the Spinal Cord."

"We shall now (say they) give some cases, in which the tenderness or morbid state of the spine is universal, that it may be seen how perfectly all its symptoms are made up of those belonging to disorder of its separate portions, and how truly they correspond in their immediate seat and nature with the situation of that point of the chain which happens to be most tender or painful. For even when there is universal soreness of spine, it will be almost always found that it is acute at a few of the vertebræ, and more or less obscure through the remainder. Besides this acute tenderness to pressure, there is also very usually a constant though variable pain, or sometimes sense of heat or burning, or mere uneasiness; and this we believe in by far the greater number of instances is felt about the seventh or eighth dorsal vertebra. This pain and the general soreness of spine are very indicative of a tedious and troublesome complaint. The most acute soreness is usually about the situation of the pain; and when the former shifts up or down the column, the pain, although it may not accompany it, generally ceases for the time. The pressing symptoms will in fact be found always more directly connected with the acute tenderness on pressure than with the pain, which, in many instances, attended with strange and variable affections, is yet never complained of, except at the seventh or eighth dorsal vertebra. There is also, in these cases, a frequent disposition to relapse, without any very obvious cause, after considerable improvement; and often, when we have relieved the irritation at one point of the spine, we shall find it has merely been transferred to another, as in the metastasis of gout or rheumatism—a tolerable proof of its frequent dependence on a general morbid disposition in the cord, and of the inutility in such instances of mere local applications, except as occasional adjuvants in the cure. As the first case detailed in this work is more strikingly illustrative of all the possible effects which it would seem the state of the cord we are describing might produce, than any other we could bring, we shall now select from our notes such chiefly as present some unusual or remarkable features. Among these, perhaps the most interesting are the instances in which sudden insensibility is brought on by pressing a particular spot in the spinal column." 147.

Many cases are adduced; but we will select a very long one, which we shall greatly condense.

*Case* 22. A pale and delicate lady, aged 40, was affected, previously to a confinement, with general œdema to an alarming degree, accompanied by head-ache, palpitation, oppression, and tightness of the chest, &c. These symptoms were relieved by blood-letting, at the close of 1828. She had a dangerous state of sinking in her confinement, and complained of a pain shooting from the back to the sternum when she put the child to the breast. The child was weaned, and the recumbent posture was enjoined, and gave relief.

Tenderness on pressure at the 7th or 8th dorsal vertebra, especially at the left side. Leeches and blisters did not succeed. The pain afterwards shifted from place to place, coming on in the evening, and continuing till midnight. Weeks passed in this way, and the symptoms took on a great variety of seat and character, corresponding with increase of tenderness in a greater extent of spine. Fits of insensibility often followed throbbing head-aches, flushed face, &c.

"In the latter end of the Spring, a new train of symptoms set in. After a slight inflammatory attack of lungs, for which some blood was taken, she became affected with a sensation as if a hair, or something of the kind, was stretched across the throat, which, after a time, grew very distressing. It was accompanied by a short troublesome cough, and eventually by a feeling of enlargement of the tongue and palate. This was suddenly followed by extreme difficulty of swallowing, with constant anxious efforts and convulsive action of the muscles of the throat; respiration became shrill, croupy, and difficult; and, when the fit reached its height, seeming imminently to threaten suffocation, she fell back in a state of quiet insensibility, out of which, after some minutes, she sprung up to repeat the paroxysm." 155.

The above is a specimen of one of the forms which the Proteian malady of this poor lady assumed. In the course of a long illness, she seems to have exhausted Cullen's or even Good's Nosology—leaving very few of the long black catalogue untouched. The worst of it was that, though every remedy in the Pharmacopœia was tried, not one of them gave permanent relief, and very few of them afforded even temporary advantage. Although there is almost a certainty of recovery in the ordinary run of such cases, it requires unusual confidence in the powers of Nature, and no very limited range of experience to allay the apprehensions of ourselves or the friends of the patient in such protracted illnesses as that now described. It is curious that one side of this lady's body was always colder than the other. The periodic head-ache by which she was occasionally affected, came on gradually at a certain hour in the morning, increasing till it became unbearable. On some occasions, when the hour arrived, the pain struck through the temple, like an electric shock, in its utmost intensity at once. Carbonate of iron arrested the head-ache, but changed it to a total deafness that came on at the exact hour which the head-ache had formerly observed.

### CHAP. VI.

*Spinal Irritation resembling Inflammatory and Febrile Affections.* The following case, which occurred in the clinical ward of the Edinburgh Royal Infirmary, strongly attracted the notice of the writers. A young woman was brought in with supposed inflammation of the bowels, for which she was bled very largely, and took large doses of opium, with little alleviation of symptoms. On the second or third day hysteria supervened, and then the physician changed his plan with advantage. In his clinical lecture, he stated that hysteria sometimes imitated inflammation so perfectly, that it was impossible to distinguish between them. He recommended his hearers, however, to treat them as inflammatory at the beginning, and afterwards, if hysterical symptoms appeared, to change the system. We do not quite agree with the Edinburgh physician as to the extreme difficulty or impossibility of discriminating nervous from inflammatory complaints. There is generally a character about the complaint and the patient which soon discloses to the observant physician the true nature of the malady. No persons, as our authors justly observe, are so liable to these attacks of pseudo-inflammation, as hysterical females after parturition. So susceptible is the uterine system, in these cases, that great injury is, no doubt, done by depletion to a great extent, under the idea of having hysteritis instead of hysteralgia to manage. We shall make room for two short cases.

*Case* 23. "Mrs. O'B——, aged 30 years, in four-and-twenty hours after a good delivery, was attacked with acute pain and tenderness in the uterine region; with frequent pulse, hot skin, and thirst. There was great pain of back, and bearing down, no expulsion of coagula, and partial suppression of the lochia. The affection was not preceded by any obvious rigor. There was excruciating tenderness of the lumbar vertebræ and sacrum on pressure."

*Case* 24. "Mrs. A. C——, in twenty-four hours after an easy delivery, was seized with a violent shivering fit, followed by acute pain and tenderness in the uterine region; with frequent hard pulse, hot skin, white tongue, and excessive thirst. There was also severe pain of back and bearing down, occurring in violent fits, without expulsion of coagula, and attended by great depression and weakness in the intervals of ease. The tenderness of the lower part of the abdomen was so great, she could scarcely bear the gentlest touch; but she could stretch down her limbs freely. The lochia were suppressed. There was most excruciating tenderness of the lumbar vertebræ and sacrum.

Both these cases were speedily relieved by fomentations to the abdomen and back, by purgatives, and by opiates with diaphoretics." 165.

Our talented friend, Dr. Marshall Hall, has laid down excellent rules for the guidance of young practitioners on occasions like the present.

### Chap. VII.

The Seventh Chapter embraces some remarks on diseases resembling those already detailed, but unattended by spinal tenderness—and probably dependent on irritation of the sympathetic ganglia. The deductions from facts in this chapter rest chiefly on hypothesis, and therefore we shall not dwell upon it. The following passage conveys the pith of the chapter.

"We should perhaps involve ourselves in as little error as is possible with our present limited information on the subject, if we merely assumed that the sympathetic ganglia must be, like all other parts of the nervous system, subject to disorder; but that as they are beyond the reach of examination, and their disorders are denoted by symptoms very analogous to similar affections of the spinal marrow and sometimes of the brain, we can only be assured of their existence by the negative evidence, that these parts of the nervous system are unaffected. Cerebral affections, however they may sometimes resemble those of the cord or ganglions, or of individual nerves, have very usually some one or two symptoms peculiar to them. And those morbid states of the spinal marrow so frequent in hysterical subjects, which are most liable to be confounded with disorders of the brain or ganglions, are generally denoted by a considerable disturbance of the nerves of relation, as well as those of organic life, and always by spinal tenderness. This last symptom can only by mere assumption be considered an effect of ganglionic disease, while we have direct proof of its absolute dependence occasionally on some disturbed state of the origin of the spinal nerves, in the facts that in some cases there is no disturbance of any of the viscera, no disorder whatsoever of the system, except what relates to certain of those nerves; and in others the tenderness is the obvious consequence of injury done to some part to which nerves from the spinal cord are distributed. The absence of this tenderness therefore, viewing it as a symptom proper to functional affections of the spinal marrow, gives us tolerable assurance that it is exempt from disorder. Whether the complaints belong to the nervous system at all, we cannot from other considerations be at a loss to determine. The neuralgiæ of either class, according to M. Jolly, have in common a preceding malaise, nausea, chilliness, some præcordial anxiety, an accompanying irritation, and an afflux of blood, with or without febrile action, a determination in perspiration or sedimentous urine, and finally intermissions between the paroxysms. In both classes of nerves, he says, the diseases yield to the same therapeutic means, and generally leave no cognizable trace after death, either in the nerves themselves, or in the organs to which these nerves are distributed." 172.

The cases in illustration are doubtful, and we shall pass them over.

### CHAP. VIII.

Chapter Eight is very short, and adduces a few cases of acute spinal inflammation. Some of these, and of an alarming character, our authors have been inclined to look upon as of a rheumatic nature—because, in all the cases that occurred to them, there was eventual recovery, however slow, without depletion of any extent. We cannot doubt, indeed, that the membranes of the cord are subject to rheumatic inflammations, in common with the serous or mucous membranes of other parts. One short case may be adduced.

*Case* 25. "A young lady was attacked with excruciating pain at the lower dorsal and upper lumbar vertebræ, increased at intervals to a most dreadful degree. She could not move her lower limbs, and suffered much when they were moved by others. She had difficulty and pain in passing water; her skin was somewhat warmer than natural; her tongue white and moist; but the pulse was very little accelerated. She recovered perfectly, in about three weeks, by the use of fomentations, purgatives with colchicum, opiates, and stimulating liniments. She was not blistered. The lady's friends were much alarmed in this case at the total paralysis of the lower limbs." 181.

The tabular view of 148 cases of spinal irritation, including all its forms, follows, and is, of course, insusceptible of analysis. It constitutes, however, a very valuable portion of the work, which will be often referred to, as giving a kind of *coup d'œil* of the whole.

The chief facts and inferences are embodied by the authors in sixteen propositions, which we shall here extract, as they cannot be abbreviated.

"1st. That tenderness at one or more points of the spine is an attendant on almost all hysterical complaints; on numerous cases of functional disorder, where the hysteric disposition is not so obvious; and in many nervous or neuralgic affections.

2d. That many of the symptoms of these affections evidently depend on a peculiar state of certain nerves, probably at their origin, may be reproduced at any moment by pressure, and are often relieved by remedies applied there.

3d. That in all the cases of tenderness of the cervical and upper dorsal spine, there was nausea, or vomiting, or pain of stomach, or affections of the upper extremities; but no pain of the abdomen, dysury, ischury, hysteralgia, or affections of the lower extremities.

4th. That in all cases of dorsal tenderness, pains affecting the abdomen, bladder, uterus, testes, or lower extremities, were usual symptoms: while nausea, vomiting, or affections of the upper extremities, were never complained of.

5th. That nausea and vomiting appeared to bear more relation to tenderness of the cervical spine—pain of stomach to tenderness of the dorsal; but that where there was soreness of both, nausea or vomiting was still more frequent, and pain of stomach scarcely ever absent.

6th. That where several points, or a great extent of the spinal column, is painful or tender on pressure, local remedies are generally less effectual, and there is a strong disposition to transference of the disordered action from one organ to another; the pain or tenderness, in all such cases of transference, shifting its place to a corresponding part of the spinal column, leaving the original point free, or with a very diminished degree of tenderness.

7th. That spinal tenderness is seldom or never met with in cases of pure inflammation, except when these accidentally occur in persons previously suffering from irritation of the cord; and that when appearances of inflammation present themselves in any organ, accompanied by a corresponding spinal tenderness, they cannot commonly be removed by the remedies applicable to inflammatory cases, and are often rendered worse by them.

8th. That there does not appear to be a complaint to which the human frame is liable, whether inflammatory or otherwise, which may not be occasionally imitated in disturbed states of the cord; and hence that this disturbed state is one vast source of those complaints called hysterical or nervous.*

9th. That those functional disorders connected with spinal tenderness are very often attended by some disturbance of the functions of the uterus; but that they are by no means always so, since they occur in those who are regular in this respect, in girls long before the menstrual period of life, in women after it has passed, and lastly in men of nervous susceptible habits, and in boys.

10th. That, in fact, they are not necessarily dependent upon the disorder of any one organ; since they are found indifferently co-existing with disturbance of the digestive organs solely, or the uterus solely, or of the circulating or respiratory system.

11th. That, from the cases detailed, we have reason to suppose spinal tenderness may arise from uterine disorder, from dyspepsia, from worms in the alimentary passages, from affections of liver, from mental emotions, from the poison of typhus, from marsh miasmata, from erysipelatous, rheumatic, and eruptive fevers, and from the irritation arising from local injury.

12th. That it is almost invariably found in connexion with gastric or abdominal tenderness in fever; and this tenderness is probably like the soreness of scalp, pains in the limbs, &c. dependent on the morbid state of the cord.

13th. That whether in fevers or in other complaints, it is met with in the situation of the eighth or ninth dorsal vertebra much more frequently than at any other part of the spine.

14th. That affections attended by spinal tenderness are seldom fatal: that

---

* Dr. Hall has here a strong support for his valuable observations on the "MIMOSES."—*Ed.*

even in those instances of intense irritation of the cord, under which patients suffer extremity of pain for years, the event is generally favourable.

15th. That they frequently, as well as hysteria, occur with all the appearances of a primary affection of the nervous system.

16th. That affections are occasionally met with, presenting all the marks of the hysteric character, and perfectly resembling cases described as those of spinal irritation, but unattended by spinal tenderness, or any other direct indication of a morbid state of the cord." 203.

From a long chapter entitled "Concluding Observations," we can only make room for a single extract, exhibiting, as it were, an epitome or condensed description of this extensive class of human ailments.

"Irritation of the spinal cord attacks persons of either sex and of all ages, but especially females at or after the age of puberty. Those of the hysteric temperament are by far more disposed to it than others. It occurs or commences in a variety of ways, and in whatever shape or degree it affects a person, may exist for a considerable time without any remarkable change. It sometimes declares itself by simple pain affecting the branches of a single pair of nerves, generally in the right or left side beneath the mamma. In such cases it seems very analogous to nervous tooth-ache, or chronic rheumatism; occasions little disturbance of the general health, and abates or recurs like these with the changes of the weather. It is very often, as Dr. Brown of Glasgow has described it, a wearying numbness rather than pain, or a sensation as if a walnut or other hard substance was pressed within a tight belt. Sometimes it begins in the right hypochondrium, extending usually to the shoulder and arm, as in complaints of the liver. At other times it supervenes on a slight inflammatory or bilious attack, and is ushered in by cough and oppression, or pain, or fever and vomiting, or by paroxysms of hysteria, faintings or palpitations. Nervous symptoms very soon appear in the greater number of these cases, however they commence, or whatever complaint they simulate. The heart, the vascular or respiratory system, become affected. We have lownesses, fits of crying, or a disposition to it from very trifling causes, with languor and debility. There is occasionally a coldness of the extremities, or of the whole person, a chilliness sometimes amounting to actual rigor, or perhaps the patient complains of odd or anomalous affections, which may not appear to have the remotest connexion with the original complaint. The pulse becomes quick and irritable, or may have been so from the commencement, and the tongue furred; two symptoms very indicative of an obstinate and troublesome attack. The stomach, the bowels, or the uterus, are occasionally affected in various ways; there is pain or pyrosis, constipation or diarrhœa, obstruction of the menses or menorrhagia, or there may be disturbance of the bladder; and these complaints frequently in the same person alternate with each other, or with disorder of the lungs or heart; but whatever the nature of the complaint may be, it is usually worse at the catamenial periods. In the severe cases epileptic fits sometimes take place, but more commonly the patient is seized with a degree of insensibility, a kind of cataleptic trance, in which all external objects are lost to her, and she is only conscious of intense pain, with throbbing or rushing of blood to the head, and perhaps sickness of stomach. When recovered from this, the state of nervous irritation is at times quite indescribable. We have heard patients complain that the slightest touch thrilled through the whole frame, or that every half inch of the surface of the body felt as if pinched or twisted, or as if screws were turning in it. Pains in the extremities, and especially in the joints, are very usual. When severe they are often supposed to arise from rheumatism; and there is generally some loss of muscular power in the upper or lower limbs, as the upper or lower portion of the cord may chance to be affected.

This is a general description of the complaint, but it will not always enable us to distinguish cases of spinal irritation from those of organic disease, which are often attended in delicate or nervous habits by many of the symptoms enumerated. Those more particularly diagnostic of irritation of the cord are,

1st. The pain or disorder of any particular organ being altogether out of proportion to the constitutional disturbance.

2ndly. The complaints, whatever they may be, usually relieved by the recumbent position, always increased by lifting weights, bending, stooping, or twisting the spine; and among the poorer classes, often consequent to the labour of carrying heavy loads, as in drawing water, manure, &c.

3dly. The existence of tenderness at that part of the spine which corresponds with the disordered organ.

4thly. The disposition to a sudden transference of the diseased action from one organ or part to another, or the occurrence of hysterical symptoms in affections apparently acute.

5thly. Perhaps we may mention the occurrence of continued fits of yawning, or sneezing. They are not very common symptoms; but as scarcely ever occurring in acute or organic diseases, they may generally be considered as characteristic of nervous irritation.

Of all these symptoms, the increase of pain on lifting weights, and the spinal tenderness, are the least equivocal. Pain of stomach, when dependent on, or connected with tenderness of spine, may always be increased by placing a weight on the head, or lifting one. The female peasantry in this country, usually complain of a great aggravation of their sufferings from carrying cans of water; and sometimes the morbid sensibility of the nerves is so extreme, that we are told, when they step inadvertently on a pebble, such sudden pain of stomach seizes them, that they feel as if life would leave them.

With regard to the spinal tenderness, we shall find on examination, there is something peculiar about it. The symptom is of course common to irritation and to a much more serious disease of the cord, but in the former it is more acute, especially in its early stage, than it is in the latter; and we sometimes find, as soon as the finger reaches the affected vertebræ, that the patient springs as if an electric spark had passed through her, or falls into a fit of insensibility, or syncope." 215.

### Chap. X.

Although the Ninth Chapter was headed "concluding observations," we find a Tenth Chapter, and a very long one, succeeding the conclusion, and entitled "Treatment." This little peculiarity in the arrangement, is merely indicative of the side of the Channel on which the work was written: and is not adduced as any blot on the performance. It might have led some of our nimble-fingered critical reviewers, however, into a scrape—especially those who review by the headings of chapters, and who seldom undergo the drudgery of reading a work from beginning to end, before the process of criticism commences.

### Treatment.

After all the views and reviews of "spinal irritation" which we have read in the work before us, and extracted for the edification of our readers, we certainly were not prepared for a second edition of "concluding observations"—and still less for the following astounding announcement:—"We shall simply review the history and nature of the complaint, and offer some observations which may, we hope, lead to a judicious mode of treatment." 221. There can be no necessity for us to review those various points which are already before our readers, and we find it rather difficult to condense the views of our authors, since they are not enumerated with much order or clearness. They class spinal affections partly according to the nature of the individual constitution—the exciting causes—and the *degree* of the malady. The distinction of cases runs thus, and is nearly the same as that adopted by Dr. Brown, of Glasgow—namely,

1. "Cases of pain affecting a single nerve, with tenderness at a corresponding part of the spinal column, and little or no constitutional disturbance.

2. Cases of a more complex nature, with tenderness of the spinal column to a greater extent, and continued symptoms of disorder in the digestive or uterine, or sometimes in the cerebral functions.

3. Cases of a similar description, but in which the disturbance of function in the different organs appears subsequently to other manifestations of the disease, or exists evidently as a secondary affection. These are chiefly the instances in which we observe a frequent metastasis of the diseased action from one set of organs to another." 223.

The neuralgiæ of the first class often continue a long time, without materially affecting the general health. The disorder is local, and is usually relieved by local remedies—namely, by leeching, blistering, &c. When, however, pain in the side or ensiform cartilage is excited by pressure on the spine—or this irritation is still kept up by pyrosis, worms, mental anxiety, or the like, local remedies will not be sufficient. Even where the complaint persists from habit, general means will be necessary in addition to local ones. This is the more necessary to be remembered, since many people have come to the conclusion that spinal irritation did not exist because it did not give way to topical remedies. Besides, spinal irritation may owe its origin to disorder or irritation in some other and even distant organ. Sir B. Brodie justly remarks that, in these latter cases, we cannot expect permanent benefit to arise from applications made to the part referred to by the patient, since the cause may be at a distance.

"If you would cure your patient," says Mr. Brodie, "you must, in each individual case that comes before you, study the disease pathologically. Endeavour to trace the symptoms to their true origin; and if you can succeed in doing so, you will in many instances learn at the same time in what manner a cure is to be effected; while in others, in which the disease does not admit of a cure, you will learn this also—to avoid tormenting your patient with useless remedies; and at any rate you will be satisfied you can do as much for him as your neighbours." 225.

Recent cases, however, are generally easy of cure. When of only two or three weeks' standing, they are sometimes perfectly relieved by an active purgative, as is the case with hysteria. The most perplexing of neuralgic pains is that of the stomach, with or without pyrosis. It is occasioned by a variety of causes, and takes place in so many different states of that organ, that it is very puzzling. In some people it comes on when the stomach contains food—in others, when it is empty—in a third class, it is most troublesome while the chyme is passing through the pylorus into the duodenum—or while undergoing the process of digestion in the second stomach. It is, as our authors justly observe, exceedingly difficult to distinguish the gastrodynia dependent on spinal irritation, from that which is occasioned by irritation of the gastric nerves themselves—and either of these from subacute or chronic gastritis. Stomach-disorders and pain are exceedingly prevalent among the Irish peasantry—partly from the cold and flatulent nature of their vegetable diet—partly from the consumption of whiskey—and partly, we may well believe, from the triste moral emotions produced by want, discontent, and political agitation. Be this as it may, our authors were very unsuccessful in their dispensary practice, as the miserable patients had no means of assisting the remedies by proper regimen.

"Out of sixty-nine cases of affection of the cervical and dorsal portion, there was pain of stomach in fifty-seven. Indeed there is no part of the spinal marrow that seems to be the centre of such general sympathy, as about the situation of the eighth dorsal vertebra. If a nervous or hysterical woman hears unfortunate news, if the catamenial flow is interrupted, or if the uterine action in advanced pregnancy becomes too powerful for the system, we believe there is no part so readily affected as the centre of the dorsal spine, no complaint so usual as the concomitant pain of stomach. 227.

In cases of simple gastrodynia, with spinal tenderness, it will always, they remark, be proper to try the effect of local treatment—leeching and blistering. The same to the epigastrium was often successful, after it had failed in the other way. After enumerating various medicines which they tried in gastrodynia with but little advantage, they announce a popular one, which exceeded in efficacy the regular prescriptions of the physician.

"But all these remedies have seemed very inferior in efficacy to a popular one among the poor in this country, which we have fallen upon by accident—the super-sulphate of alumen. We first saw it used in the case of a patient afflicted with pain of stomach, sometimes occurring in violent paroxysms, and accompanied by vomiting and pyrosis. There was great tenderness at the pit of the stomach, and in the right hypochondrium. The complaint had subsisted long, and he had been under a variety of treatment with little benefit. It was, in fact, eventually supposed to depend on serious organic disease of the liver and of the stomach. About this time, however, he was prevailed on, by a friend of his, to take an ounce of alum in a dose. It acted as a purgative, and gave such immediate relief, that he was induced to repeat it. The benefit he again experienced was very considerable; and, by persevering in the remedy, a cure was eventually effected." 229.

They are now in the habit of prescribing a tea-spoonful of alum powder, twice a day, with two aloetic pills every night, with great benefit to their patients—"acting, in some instances, like a charm, on a state of disorder which has resisted other remedies for years."

"The success with which these and other similar medicines are occasionally exhibited in cases of gastrodynia and pyrosis, accompanied by tenderness in the epigastrium, gives us a tolerable assurance that they are not always, nor even commonly, dependent on any inflammatory state of the mucous membrane. They are evidently connected with disordered functions of the nerves." 229.

Where a larger portion of the spine is affected, and various organs deranged in consequence, we must act according to the nature of the case. In respect to blisters, "they should be long and narrow, so as to cover many of the affected vertebræ." The preternatural sensibility of the nervous system is to be obtunded by narcotics—especially by henbane and belladonna—the latter as a plaster to the spine, or to the suffering organ. The recumbent posture they conceive to be quite unnecessary in the generality of cases —and, in many instances, injurious.

"Of all the remedies which we have made use of, in severe cases, mercury was certainly the most successful. We were first induced to try it from its beneficial effects in instances, where spinal irritation was mistaken for a liver complaint, to two of which we may refer. It will be perhaps a matter of dispute whether in these, an affection of liver did not really exist as the primary malady; but we believe it will be found, that mercury frequently possesses some specific influence over disorder of the cord, whether connected with disease of liver or not. The hard, loud, hacking cough, which is so often a troublesome symptom, will sometimes yield to a mild mercurial course where every thing else has failed. We regret that our experience of its effects has been as yet too limited to permit our recommending it as very generally applicable; but even if it should prove so, it ought to be held in remembrance, that there are many constitutions either so enfeebled or peculiar, as totally to preclude its employment." 240.

Of all remedies, however, which prove influential in removing irritation, those which act through the medium of the mind are probably the most powerful and sudden.

"The magical effects sometimes witnessed on change of air, are, we believe, for the most part attributable to the influence of change of scene and place on the mind; and are only to be equalled by the extraordinary amendments or recoveries which occasionally take place from mere mental emotion.

We have seen a patient, weak and emaciated, harassed with cough, despaired of by her friends, and deriving no relief for months that she was under the care of experienced physicians, recover, as if by charm, on removing a few miles into the country. What, we may ask, were the agents in so sudden and so wonderful a cure?—sensations that the heart showed no consciousness of, and ideas impressive only from their novelty! There were no powerful excitements, no violent passions or emotions in operation, nothing but the quiet and agreeable, the silent working of new feelings, new trains of thinking; and the result of this diversion of the mind from its former depressing reflections, diminished sufferings and renovated hopes." 243.

From a more extended sphere, as well as period of observation on this point, we think we can safely say that our authors have overrated the moral, and underrated the physical effects of change of air in chronic ailments generally. We have seen the beneficial effects of this change on health, as sudden and great in the most obtuse intellects, as in people with the most sensitive feelings and impressionable imaginations—nay, often more so in the former than in the latter class. This could hardly have been the case, had the body been solely, or even principally, influenced through the medium of the mind. By this we do not mean to disparage the powerful agency of the soul on the body: we only insist on the physical agency of change of air on health, as one of very potent influence.

---

We have now brought a long, faithful, and, we hope, useful analysis to a close, seldom intruding our own observations or reflections, lest the thread of the subject should be broken, and the views of the authors interrupted. If the investigation be important, as we are sure it is, we have aided that investigation by diffusing the main facts and features of the work through an infinitely wider circle of practitioners than they otherwise would visit in the book itself. We should not have bestowed this labour on a work of inferior utility—and we trust that we have done both the authors and our readers justice in our task. It is no disparagement to the *former* to consider them as men, and liable to err. It is not unusual for men of the greatest talent and integrity to become, as it were, enamoured of the subject of their investigation, and like all lovers, to be a little blind to the failings of their favourite. It is not improbable that this has been, to a certain extent, the case with Dr. and Mr. Griffin. They may have seen, or rather fancied, spinal irritation, where it did not exist, or where it only formed part of general nervous irritability, especially in females. We all know that a distinguished physiologist and physician *felt* or fancied tenderness of the epigastrium in three-fourths of his patients, and inferred from this *fact* that chronic gastritis was their complaint. Other practitioners followed in the same path of investigation, but less biassed in favour of a theory, and soon discovered that this epigastric tenderness very often existed in people who were in perfect health—and that, in comparatively few, was it a criterion of inflammation in the subjacent organ. So it may turn out with tenderness of the spine. Even since the commencement of this analysis, we have taken several opportunities of examining the spine, and, in more than one or two instances, found patients complain of tenderness in certain portions of it, where there was not the slightest symptom to indicate irritation there, nor to suspect its existence at all,—their complaints being totally unconnected with such class of maladies. This phenomenon is not easily accounted for. On the other hand, we this very day (first of November) traced a complicated and anomalous train of symptoms to spinal irritation, before unsuspected. When we pressed steadily on a certain portion of the spine, the most acute pain was instantly felt in the epigastric region; and this was proved to be no deception by repeated examination of the whole column. We therefore conscientiously recommend the investigation to the serious attention of all our brethren.

